# Supplementary material for: The Importance of Genetic Testing in the Differential Diagnosis of Atypical TSC2-PKD1 Contiguous Gene Syndrome—Case Series
Source: Children (Basel). 2023 Feb 22;10(3):420. doi: 10.3390/children10030420 (PMC10047143; doi:10.3390/children10030420)
Supplement: Supplementary file 1 [file children-10-00420-s001.zip › children-2238732-supplementary.pdf]

Supplementary Material

Table S1. Summary of the confirmed mutations of the four patients

	**Mutation**	**Deleted exons**
Patient 1	*TSC2-PKD1* del het (de novo)	*TSC2*: 17-42; *PKD1*: 1-46
Patient 2	*TSC2-PKD1* del het (de novo)	*TSC2*: 14-42; *PKD1*: 11-46
Patient 3	*TSC2-PKD1* del het (de novo)	*TSC2*: 22-42; *PKD1*: 1-46
Patient 4	*NTHL1-TSC2-PKD1* del het (de novo)	*NTHL1*: 1; *TSC2*: 1-42; *PKD1*: 1-46

Table S2. A summary of the symptoms of our patients considering the updated diagnostic criteria of tuberous sclerosis

**Diagnostic criteria**	**Patient 1**	**Patient 2**	**Patient 3**	**Patient 4**
**Major criteria**				
Hypomelanotic macules (≥3; at least 5 mm diameter)	+	+	+	+
Angiofibroma (≥3) or fibrous cephalic plaque	+	-	-	+
Ungual fibromas (≥2)	-	-	-	-
Shagreen patch	+	-	+	-
Multiple retinal hamartomas	+	-	-	-
Multiple cortical tubers and/or radial migration lines	+	+	+	+
Subependymal nodule (≥2)	-	+	+	+
Subependymal giant cell astrocytoma	-	-	+	-
Cardiac rhabdomyoma	-	+	+	+
LAM	-	-	-	-
Angiomyolipomas (≥2)	+	+	-	+
**Minor criteria**				
"Confetti" skin lesions	+	+	-	-
Dental enamel pits (≥3)	-	-	-	-
Intraoral fibromas (≥2)	-	-	-	-
Retinal achromic patch	-	+	-	+
Multiple renal cysts	+	+	+	+
Nonrenal hamartomas	+	-	-	-
Sclerotic bone lesions	-	-	-	-
